# Weight management in overweight or obesity: Implications for cancer pathogenesis and prognosis^☆^

**DOI:** 10.1016/j.cpt.2025.05.001

**Published:** 2025-05-09

**Authors:** Yue Wang, Haitao Niu, Peng Lyu, Bing Liu, Junmin Wei

**Affiliations:** National Institutes for Food and Drug Control, Beijing 102629, China; National Health Commission of the People's Republic of China, Beijing 100044, China; Cancer Pathogenesis and Therapy, Chinese Medical Association Publishing House, Beijing 100052, China; Key Laboratory of Knowledge Mining and Service for Medical Journals, National Press and Publication Administration, Beijing 100052, China; Key Laboratory of Knowledge Mining and Service for Medical Journals, National Press and Publication Administration, Beijing 100052, China; Chinese Medical Association Publishing House, Beijing 100052, China

**Keywords:** Body mass index, Cancer pathogenesis, Cancer prognosis, Obesity, Overweight

## Introduction

Overweight and obesity are usually measured using body mass index (BMI), with overweight classifying as a BMI of 25–29.9 kg/m^2^, obesity as a BMI of ≥30 kg/m^2^, and morbid obesity as a BMI of ≥40 kg/m^2^.^1^ Overweight and obesity are major health crises, posing a threat to the current global health progress.^2^ In 2021, 3.71 million deaths and 129 million disability-adjusted life-years (DALYs) were attributable to overweight and obesity.^3^ With ongoing surges in obesity among children and adolescents worldwide, adult obesity rates are expected to increase. Consequently, the burden of various non-communicable diseases (NCDs) – particularly diabetes, cardiovascular diseases, and cancer – will continue to escalate.^4^ The number of obesity-related cancer cases is also expected to rise to >2 million new cases globally by 2070, accounting for 7% of all cancers.^5^ Accumulating evidence indicates that weight management not only affects metabolic diseases but also influences cancer development, progression, and therapeutic outcomes. We aim to discuss recent research to explore the multidimensional relationships between body weight changes and cancer pathogenesis and treatment.

## Epidemiological association between increased body weight and cancer risk

The International Agency for Research on Cancer (IARC) Working Group identified convincing evidence that excess body weight is associated with an increased risk of cancer in at least 13 anatomical sites, including endometrial, esophageal, renal, and pancreatic adenocarcinomas; hepatocellular carcinoma; gastric cardia cancer; meningioma; multiple myeloma; and colorectal, postmenopausal breast, ovarian, gallbladder and thyroid cancers.^6^ A meta-analysis demonstrated that each kg/m^2^ increase in BMI was associated with a 1.01- to 1.13-fold increased risk of general brain tumors, multiple myeloma, bladder cancer, pancreatic cancer, breast cancer, and non-Hodgkin's lymphoma. Additionally, every 1 kg/m^2^ increase in BMI was linked to 6% and 4% increases in the risk of kidney cancer and gallbladder cancer, respectively.^7^

## Pathophysiological mechanisms

The importance of the tumor microenvironment in the development, growth, and progression of cancer is now well-established. Chronic inflammation is a classic and prevalent example of ongoing perturbation within the microenvironment. Unattended hyperadiposity commonly leads to metabolic disorders, altered production of steroid hormones, and chronic subclinical inflammation. Therefore, these pathophysiologic effects have been associated with tumor development and progression.^8^

### Chronic inflammation and immune microenvironment

The inflammatory microenvironment has three primary stages of tumor formation and development (initiation, promotion, and metastasis). The inflammatory microenvironment involves immune cells, inflammatory cells, and bioactive chemicals, such as tumor necrosis factor-alpha (TNF-α), interferon-gamma (IFN-γ), interleukin, and chemokines, which participate in cancer cell tumorigenesis, growth, angiogenesis, metastasis, and resistance to treatment.^9^ Additionally, obesity-related T-cell dysfunction has been shown to impair immunosurveillance and increase cancer risk.^10^

### Metabolic dysregulation and signaling pathways

Emerging evidence suggests a link between adipokines and epithelial–mesenchymal transition (EMT). An association has been reported between EMT in cancer and the adipokines leptin, adiponectin, resistin, visfatin/nicotinamide phosphoribosyltransferase (NAMPT), lipocalin-2/neutrophil gelatinase-associated lipocalin (NGAL), as well as other newly discovered adipokines including chemerin, nesfatin-1/nucleobindin-2, zinc-α2-glycoprotein (AZGP1), secreted frizzled-related protein 5 (SFRP), and fatty acid-binding protein 4 (FABP4).^11^

### Steroid hormones and receptors on carcinogenesis

Steroid hormones regulate cellular activities in many physiological and pathological processes in various tissues. Dysregulation of steroid hormones and receptors is closely associated with many diseases, including cancer. The role of steroid hormones and their receptors in breast cancer, endometrial cancer, and gastric cancer, the mechanisms underlying these roles, and the clinical relevance of deregulated steroid hormone receptors (SRs) have been thoroughly investigated in patients with cancer. Estrogen (ER), androgen (AR), and progesterone (PR) receptors and closely related glucocorticoid receptors (GRs) are all involved in ligand-independent phosphorylation events by proline-directed kinases in the cyclin-dependent kinase (CDK) and mitogen-activated protein kinase (MAPK) families. Faced with drugs that reliably target SR ligand-binding domains to block uncontrolled cancer growth, ligand-independent post-translational modifications guide changes in cell fate that confer increased survival, EMT, migration/invasion, stemness properties, and therapy resistance of non-proliferating SR^+^ cancer cell subpopulations.^12^

## Relation weight management with cancer therapy

### Survival and recurrence

Kalb et al.^13^ investigated a study cohort of 612 patients who underwent surgery to treat rectal carcinoma. The study showed that patients with obesity class II or higher (BMI ≥35 kg/m^2^) and patients who were underweight (BMI <18.5 kg/m^2^) had a reduced overall survival (OS) (hazard ratio [HR] = 1.6; 95% confidence interval [CI] 0.9–2.7) as well as higher rates of distant metastases (HR = 1.7; 95% CI 0.9–3.3) compared to patients with normal body weight (18.5 ≤ BMI <25 kg/m^2^) and those who are overweight (25 ≤ BMI <30 kg/m^2^) or with obesity class I (30 ≤ BMI <35 kg/m^2^).

Chen et al.^14^ showed that severe obesity (BMI ≥40.0 kg/m^2^) at baseline was independently associated with worse disease-free survival (DFS) (HR = 1.48; 95% CI 1.02–2.16; *P* = 0.040) and OS (HR = 1.79; 95% CI 1.17–2.74; *P* = 0.007) compared with underweight/normal weight (BMI ≤24.9 kg/m^2^).

Another study showed that compared with normal weight, increased body weight (overweight, obesity, and high BMI) led to worse DFS (*P* < 0.001) and OS (*P* < 0.001) in patients with early-stage breast cancer.^15^

## Weight management: A double-edged sword

### Surgical treatment

A randomized controlled trial demonstrated that a 4%–6% weight loss can help breast cancer survivors reach optimal body weight, substantially reducing circulating levels of ERs and cytokines, which are potential drivers of recurrence.^16^ However, severe weight loss (BMI <18.5 kg/m^2^) may worsen outcomes due to muscle wasting.^17^

### Pharmacological effect

Glucagon-like peptide 1 (GLP-1) agonist semaglutide reduced pancreatic tumor volume in mice models by altering stroma deposition, allowing T lymphocyte infiltration.^18^ Bariatric surgery was associated with a significant reduction in esophageal and gastric cancer incidence and overall in-hospital mortality; the HR of cancer incidence was significantly in favor of the surgical group (HR = 0.76; 95% CI 0.59–0.98; *P* = 0.030). Overall mortality was significantly lower in the surgical group (HR = 0.60; 95% CI 0.56–0.64; *P* < 0.001).^19^

### Prognostic value of obesity

Ji et al.^20^ analyzed 81 patients diagnosed with stage IVB cervical cancer, focusing on the visceral-to-subcutaneous adipose tissue area ratio (VSR) and skeletal muscle index (SMI) before treatment. The OS rate of patients with a high VSR was significantly higher than that of patients with a low VSR (*P* = 0.022), which suggests that visceral obesity may pose a protective effect on prognosis, challenging the conventional view that obesity is detrimental to cancer outcomes.

Certain studies have correlated obesity with poor outcomes, whereas others have observed that obesity benefits some cancer patients receiving immunotherapy, with sex differences playing a potential role.^21^^,^^22^ One proposed mechanism for improved responses to programmed cell death protein 1 (PD-1) checkpoint blockade is that obesity-associated leptin directly induces PD-1 expression on T cells, making them more amenable to blockade.^22^ Of course, the discussion on obesity/overweight and cancer treatment remains controversial; therefore, this hypothesis needs further research and clinical studies to achieve a definitive consensus.

## Conclusion and future directions

Fat accumulation in non-adipose tissue and inflammation induced by visceral fat are the most important causal factors for obesity-associated diseases. Decreasing the uptake of fat and carbohydrates and proper physical activity to control body weight and reduce visceral fat may help lower the risk of metabolic disorders. Given the worldwide epidemic of overweight and obesity, controlling body weight and a balanced diet are necessary. Additionally, understanding the links between increased weight and cancer, as well as the internal underlying mechanisms, could help prevent and treat both obesity and cancer. Therefore, weight management should be integrated into comprehensive cancer health management. Future research should prioritize tumor-type specific stratification and multi-omics approaches for personalized interventions.

## Author's contribution

Yue Wang and Haitao Niu performed the analyses, drafted and revised the manuscript; Peng Lyu, Bing Liu, and Junmin Wei designed and supervised this study, and critically reviewed the manuscript. All the authors have read and approved the final paper.

## Ethics statement

None.

## Declaration of Generative AI and AI-assisted technologies in the writing process

The authors declare that generative artificial intelligence (AI) and AI assisted technologies were not used in the writing process or any other process during the preparation of this manuscript.

## Funding

None.

## Data availability statement

The datasets used in the current study are available from the corresponding author on reasonable request.

## Conflict of interest

The authors declare that they have no known competing financial interests or personal relationships that could have appeared to influence the work reported in this paper.
